# Chronic inflammation-related DNA damage response: a driving force of gastric cardia carcinogenesis

**DOI:** 10.18632/oncotarget.3091

**Published:** 2014-12-30

**Authors:** Runhua Lin, Dejun Xiao, Yi Guo, Dongping Tian, Hailong Yun, Donglin Chen, Min Su

**Affiliations:** ^1^ Institute of Clinical Pathology, Guangdong Provincial Key Laboratory of Infectious Disease and Molecular Immunopathology, Shantou University Medical College, Shantou, Guangdong, PR China; ^2^ The Judicial Critical Center, Shantou University Medical College, Shantou, Guangdong, PR China; ^3^ Cancer Hospital of Shantou University Medical College, Shantou, Guangdong, PR China; ^4^ Clinical Laboratory of Ganzhou People's Hospital, Ganzhou, Jiangxi, PR China

**Keywords:** chronic inflammation, DNA damage response, precancerous lesions, genomic instability, gastric cardia carcinogenesis

## Abstract

Gastric cardia cancer (GCC) is a highly aggressive disease associated with chronic inflammation. To investigate the relationship between DNA damage response (DDR) and chronic inflammation, we collected 100 non-tumor gastric cardia specimens of Chaoshan littoral, a high-risk region for esophageal and gastric cardia cancer. A significantly higher proportion of severe chronic inflammation was found in dysplastic epithelia (80.9%) in comparison with that in non-dysplastic tissues (40.7%) (P<0.001). Immunohistochemical analysis demonstrated that DNA damage response was parallel with the chronic inflammation degrees from normal to severe inflammation (P<0.05). We found that DNA damage response was progressively increased with the progression of precancerous lesions (P<0.05). These findings provide pathological evidence that persistent chronic inflammation-related DNA damage response may be a driving force of gastric cardia carcinogenesis. Based on these findings, DNA damage response in non-malignant tissues may become a promising biomedical marker for predicting malignant transformation in the gastric cardia.

## INTRODUCTION

Gastric cancer represents the fourth most common cancer and second leading cause of cancer-related death worldwide, taking a toll of approximately 738,000 people in 2008 alone [1]. It was once considered as a single entity. Now, scientists have divided this cancer into two main types: gastric cardia cancer (cancer occurs in the top portion of the stomach near the gastro-esophageal junction) and non-cardia gastric cancer (cancer in all other areas of the stomach). The incidence of GCC has a remarkable geographic aggregation, which resembled the epidemiological features of esophageal squamous cell carcinoma (ESCC), particularly in high-risk areas [2, 3]. Our previous epidemiological study showed a high incidence of GCC (34.8/100,000) in Nan'ao Island of Shantou, an isolated Chaoshan littoral region of China, from 1995 to 2004 [2], highlighting the need for greater understanding of its pathogenesis. Nevertheless, the vast majority of cancers originating from gastric cardia are adenocarcinomas based on histopathological features. These epidemiological and histopathological characteristics confer uniqueness to GCC as a clinical entity. The gastro-esophageal junction (including the proximal gastric cardia region) is an anatomical location with a remarkably high and rapidly rising incidence of adenocarcinoma [4-6], while the underlying mechanisms for the development of GCC are poorly understood [7].

Inflammation is a vital defensive response that serves as critical roles in a variety of physiological situations, and when dysregulated, can contribute to the pathogenesis of many diseases, including cancer. Chronic inflammation is a well-documented risk factor for cancer development [8-10]. Links between inflammation and cancer were first reported by Rudolf Virchow in 1863, on the basis of observations that tumors often arise at sites of chronic inflammation and inflammatory cells were present in biopsy samples from tumors [11]. Later on, a substantial body of evidence revealed that chronic inflammation may be a causative factor of a variety of cancers. Clinical epidemiological studies revealed that over 15%-20% of all solid tumors may be related to chronic inflammation [12-15]. The question of how chronic inflammation can initiate tumorigenesis has interested scientists for decades and is far to be resolved and clarified. Therefore, there is a need for investigation on relevant mechanisms of chronic inflammation-related tumor development.

DNA damage occurs through exposure to various toxic agents, including inflammatory cytokines [16, 17]. Under chronic inflammatory conditions, reactive oxygen species (ROS) and reactive nitrogen species (RNS) are generated and result in DNA damage [18]. Based on the results from our previous study regarding DNA damage response in peritumoral regions of esophageal cancer microenvironment, herein, we focus on the possible role of chronic inflammation-related DNA damage response during the process of gastric cardia carcinogenesis.

Today, the causal relationship between chronic inflammation and cancer is more widely accepted, whereas the underlying molecular mechanisms mediating this relationship continue to be elucidated. In this current study, we therefore investigate the relationship between DNA damage response status and chronic inflammation as well as intraepithelial neoplasia of the gastric cardia. Our findings showed that DNA damage response in non-malignant tissues correlated with enhanced chronic inflammation and was also associated with progression of precancerous lesions, suggesting a pivotal role of chronic inflammation-related DNA damage response in gastric cardia tumorigenesis.

## RESULTS

### Significantly higher proportion of severe chronic inflammation in dysplastic tissues

**Figure 1:**
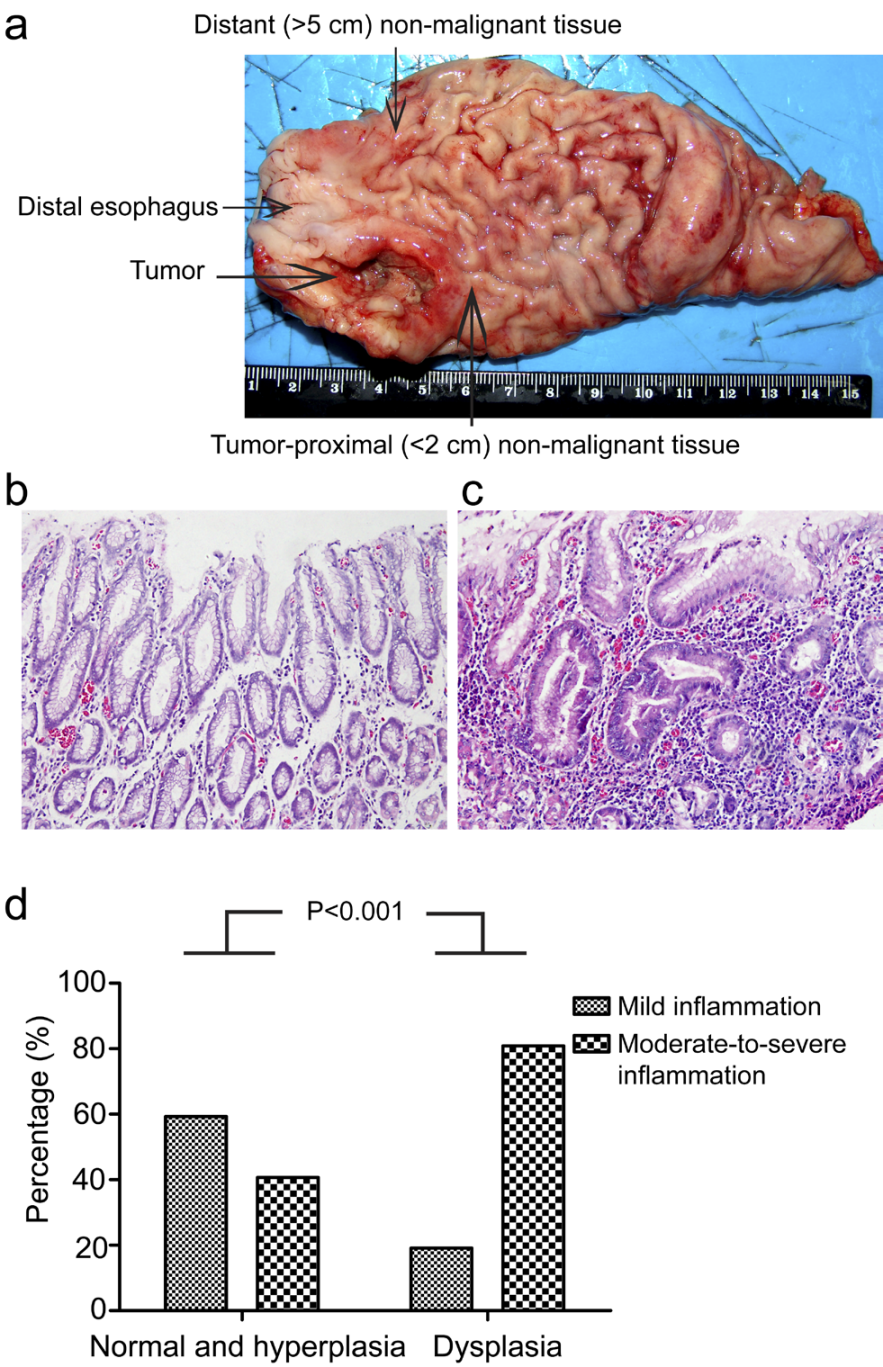
Chronic inflammation correlates with dysplasia in gastric cardia epithelia (a) Schematic illustration of gastric cardia epithelial tissue sample collection. (b) Representative image of normal gastric cardia mucosa with mild inflammation. (c) Representative image of dysplastic gastric cardia mucosa with severe inflammation. (d) Percentage of different degrees of chronic inflammation in indicated types of tissues are shown.

To clarify the possible role of chronic inflammation in gastric cardia carcinogenesis, it is of fundamental importance to evaluate chronic inflammation status in gastric cardia tissues without malignant changes. To achieve this, we examined a total of 100 non-malignant gastric cardia tissues (50 tumor-proximal non-malignant tissues and 50 distant non-malignant tissues) from 50 GCC patients by analyzing inflammation status in tissues with different pathological changes. Our results revealed that severe chronic inflammation was more frequently observed in dysplastic tissues (80.9%), such as low-grade intraepithelial neoplasia (LGIN) and high-grade intraepithelial neoplasia (HGIN) in comparison with that in non-dysplastic (normal/hyperplasia) tissues (40.7%) (P<0.001) (Figure 1b-d), indicating a potential link between chronic inflammation and gastric cardia carcinogenesis.

### DDR is parallel with the severity of chronic inflammation

**Figure 2:**
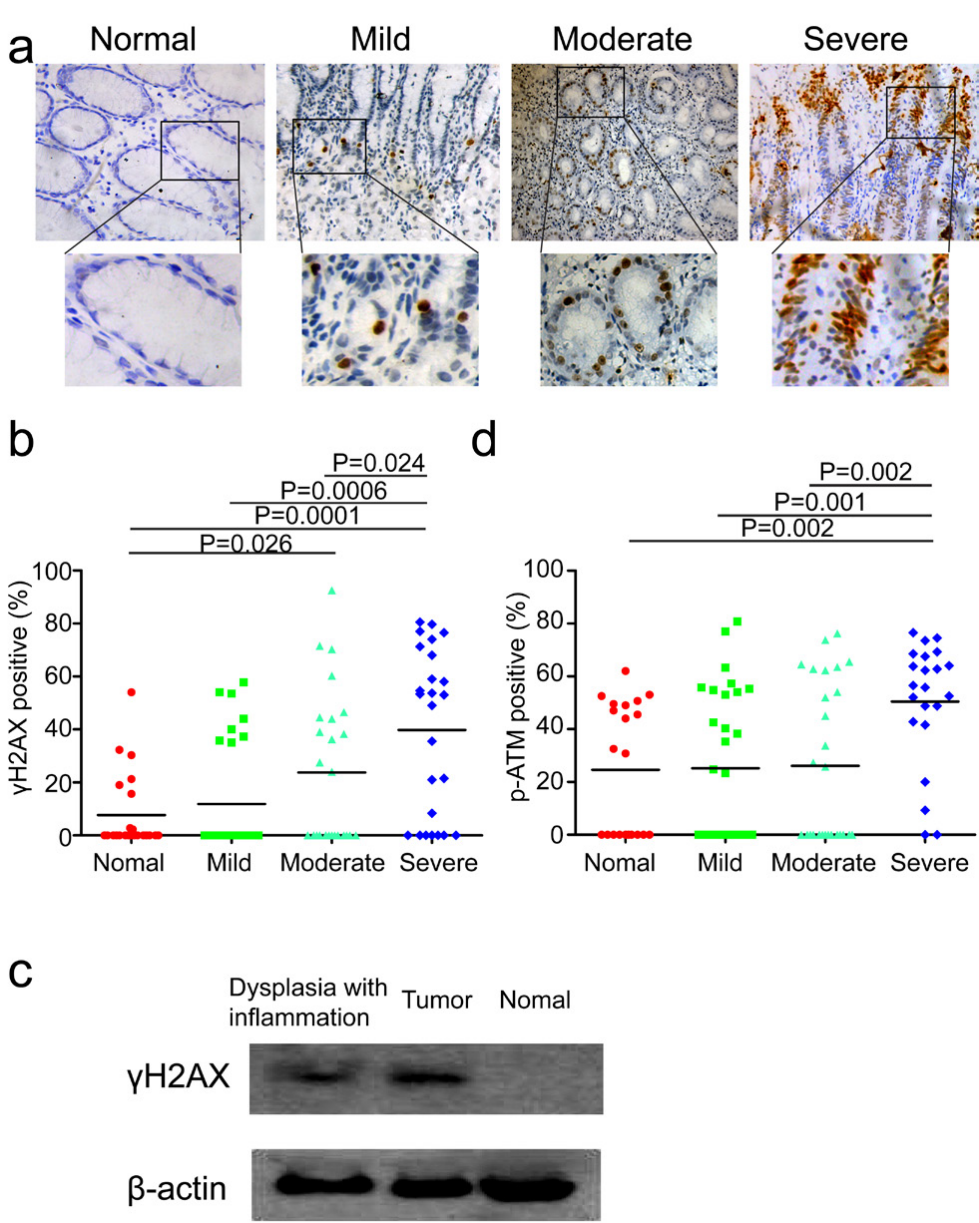
DNA damage response is associated with the degree of chronic inflammation (a) Representative images of γH2AX staining in tissues without evident inflammation, with mild, moderate and severe inflammation. (b) Quantification of γH2AX positive cells in tissues without evident inflammation, with mild, moderate and severe inflammation from 50 gastric cardia cancer patients (two non-malignant samples from each patient). (c) Representative western blot analysis of γH2AX and ACTIN in tumor tissue, inflamed tissue with dysplasia and histologically normal tissue without evident inflammation. (d) Quantification of phospho-ATM positive cells in tissues without evident inflammation, with mild, moderate and severe inflammation from 50 gastric cardia cancer patients (two non-malignant samples from each patient).

It has been previously reported that the formation of DNA lesions induced by reactive oxygen and nitrogen species (RONS) during chronic inflammation could lead to colonic tumorigenesis in mice [17]. In addition, our previous study has demonstrated that DNA damage response was increased in esophageal cancer tissues and tumor-surrounding non-malignant tissues compared with more distant tissues in human esophageal tissues [19]. Hence, we speculated that whether DNA damage serves as the intermediate to bridge the gap between chronic inflammation and gastric cardia carcinogenesis. If that is the case, DNA damage response should be more likely to be detected in inflamed tissues than tissues without evident inflammation. First, we attempted to investigate the actual status of DDR in the above tissues with various degrees of chronic inflammation. Phosphorylation of histone H2AX (γH2AX) and its recruitment are one of the very first events in indentifying damaged DNA, which is also a reliable marker for DNA double-strand breaks [20]. Not surprisingly, immunohistochemical analysis showed that different proportion of epithelial cells in inflamed tissues stained positive for γH2AX. The strongest and most abundant immunostaining of γH2AX was observed in epithelial cells accompanied by severe chronic inflammation. A less intense γH2AX signal was found in samples with mild-to-moderate inflammation, normal gastric cardia tissues without evident chronic inflammation were totally negative for γH2AX (Figure 2a). Intriguingly, quantification analysis for γH2AX revealed a significant increase of γH2AX staining in tissues with severer chronic inflammation compared to samples without evident inflammation (Figure 2b). The same results were obtained by western blot analysis of γH2AX in these tissues (Figure 2c). To further ascertain this phenomenon, we subsequently sought to measure other DDR markers. The activation of ataxia telangiectasia mutated kinase (ATM) by phosphorylation at Ser1981 is an early event at DNA damage loci as well. Consistent with the immunohistochemical results of γH2AX, phosphorylation of ATM was also detected in inflamed tissues, with stronger signal in severer degrees of chronic inflammation compared with non-inflammation tissues (Figure 2d). These results suggested that severe chronic inflammation, to some extent, was associated with higher levels of DDR in gastric cardia epithelia. Conforming to our hypothesis, elevated levels of DDR might be induced by chronic inflammation, which eventually precipitate carcinogenesis through genomic instability.

### DDR correlates with premalignant lesions

**Figure 3:**
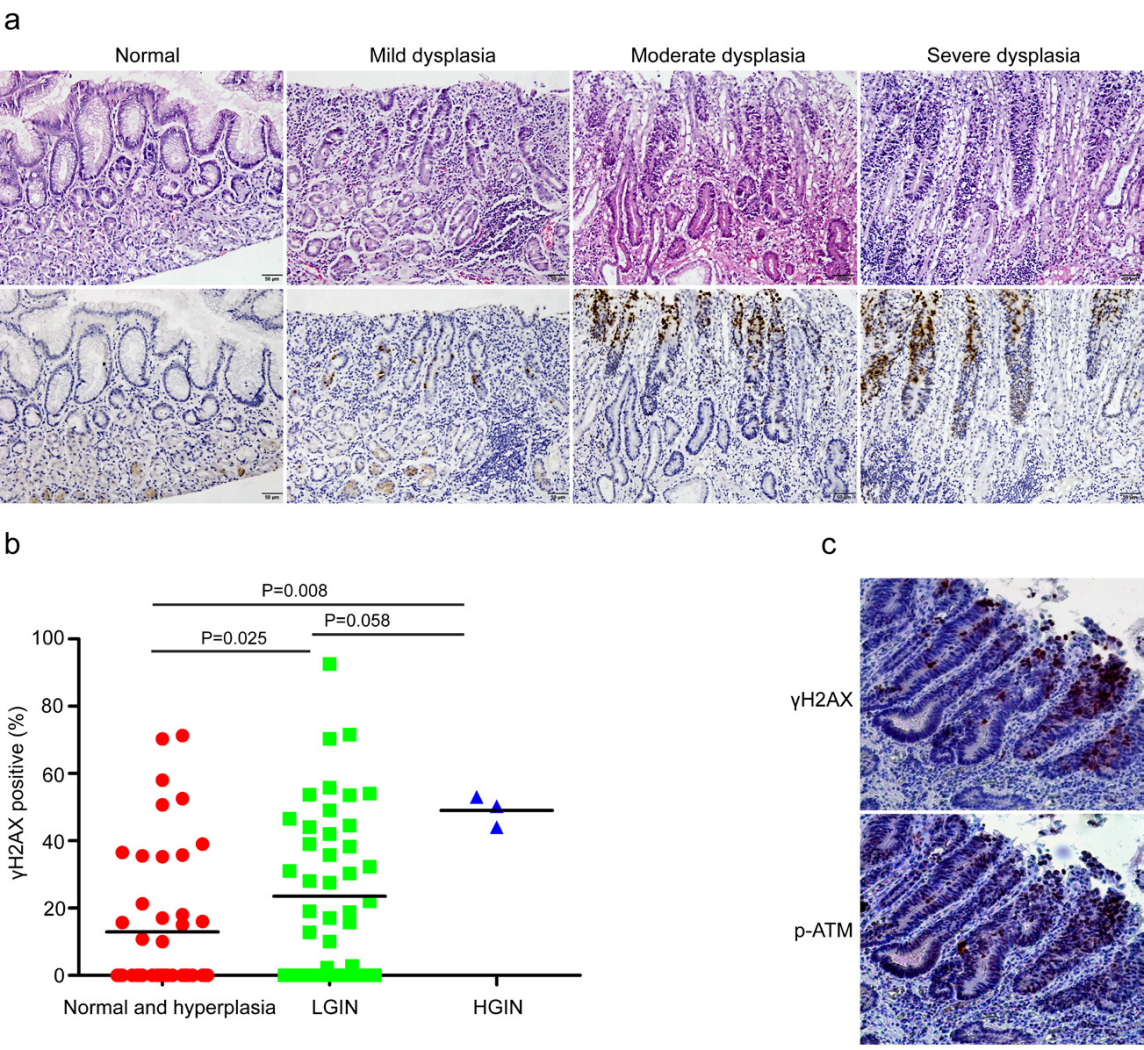
DNA damage response correlates with premalignant lesions in gastric cardia epithelia (a) Representative images of γH2AX staining in normal, mild, moderate, and severe dysplastic tissues. (b) Quantification of γH2AX positive cells in non-malignant samples grouped according to benign or preneoplastic conditions. (c) Representative images showing the co-localization of γH2AX and phospho-ATM in dysplastic tissues with chronic inflammation in the same section.

We have now unveiled the close relationship between DNA damage response and chronic inflammation. It is reasonable to hypothesize that DDR is supposed to be evident in samples with precancerous lesions in case of its participation in tumor development. It is widely recognized that chronic inflammation of the gastric mucosa can progress through sequential premalignant stages from atrophic gastritis, intestinal metaplasia (IM) and dysplasia to ultimately gastric cancer [21, 22]. It is also well-known that the development of cancer is a complicated multi-step process involving multiple genetic alterations. In fact, our previous study has confirmed that DDR levels correlated significantly with the progression stages of premalignant lesions in esophageal squamous epithelium. To test our hypothesis, we analyzed the immunohistochemical results of DDR grouped by different pathological changes in gastric cardia tissues, such as histologically-normal epithelium, low-grade intraepithelial neoplasia (LGIN), and high-grade intraepithelial neoplasia (HGIN). Not surprisingly, immunohistochemical analysis showed that the expression of γH2AX progressively increased in the sequential stages from histologically-normal epithelia to HGIN (Figure 3a&b), indicating increased genomic instability during the multi-step process of gastric cardia carcinogenesis. Additionally, we have confirmed the colocalization of γH2AX and p-ATM through performing double immunostaining in the same section (Figure 3c). These results suggested that unrepaired DNA damage in these precancerous gastric cardia tissues may be the source of its carcinogenesis.

## DISCUSSION

In this study, we used human gastric cardia tissues to investigate the association between DNA damage response and chronic inflammation as well as precancerous lesions. Firstly, we observed that dysplastic epithelial cells of the gastric cardia were frequently accompanied by severe chronic inflammation characterized by macrophage and lymphocyte infiltration, prompting us to seek the underlying mechanisms bridging the gap between chronic inflammation and gastric cardia carcinogenesis. Subsequently, immunohistochemical studies revealed that the level of DNA damage response, evident as positive staining for γH2AX and phospho-ATM, was parallel with the severity of chronic inflammation, suggesting that the emergence of DNA damage response is likely to be induced by chronic inflammation. Furthermore, we showed that DDR levels were associated with premalignant lesions, indicating increased genomic instability in the multi-step process during gastric cardia carcinogenesis. These results provide pathological evidence in human gastric cardia tissues for the critical role of chronic inflammation-related DNA damage response in gastric cardia carcinogenesis.

Gastric cardia cancer ranks the second leading malignancy in Chaoshan region, a high-risk area for ESCC and GCC in southern China. Adenocarcinomas arising from the gastric cardia share the same histopathological features with esophageal adenocarcinoma (EAC), which is a common malignancy arises from metaplastic Barrett's esophagus caused by chronic exposure to acid and bile [23]. Gastro-esophageal reflux disease (GERD) is an established etiological factor of EAC [24], whereas, the etiology of GCC remains obscure. Our results revealed that a significantly higher proportion of severe inflammation was observed in dysplastic samples (80.9%) compared to that in normal or simple hyperplastic tissues (40.7%) (P<0.001), pointing out that chronic inflammation may be an initiating factor of gastric cardia carcinogenesis in Chaoshan high-risk region. The existence of chronic inflammation in the gastric cardia may partly be elicited by *Helicobacter pylori* infection [25].

Inflammation is part of the human body's normal response to tissue damage inflicted by infections or other stimuli, when it becomes chronic, however, it can cause various pathologies including cancer [26-28]. Previous studies revealed that certain malignancies arise at sites affected by severe chronic inflammation [29, 30]. Actually, chronic inflammation might not contribute to carcinogenesis directly, since not all chronic inflammation ended up with cancer.

**Figure 4:**
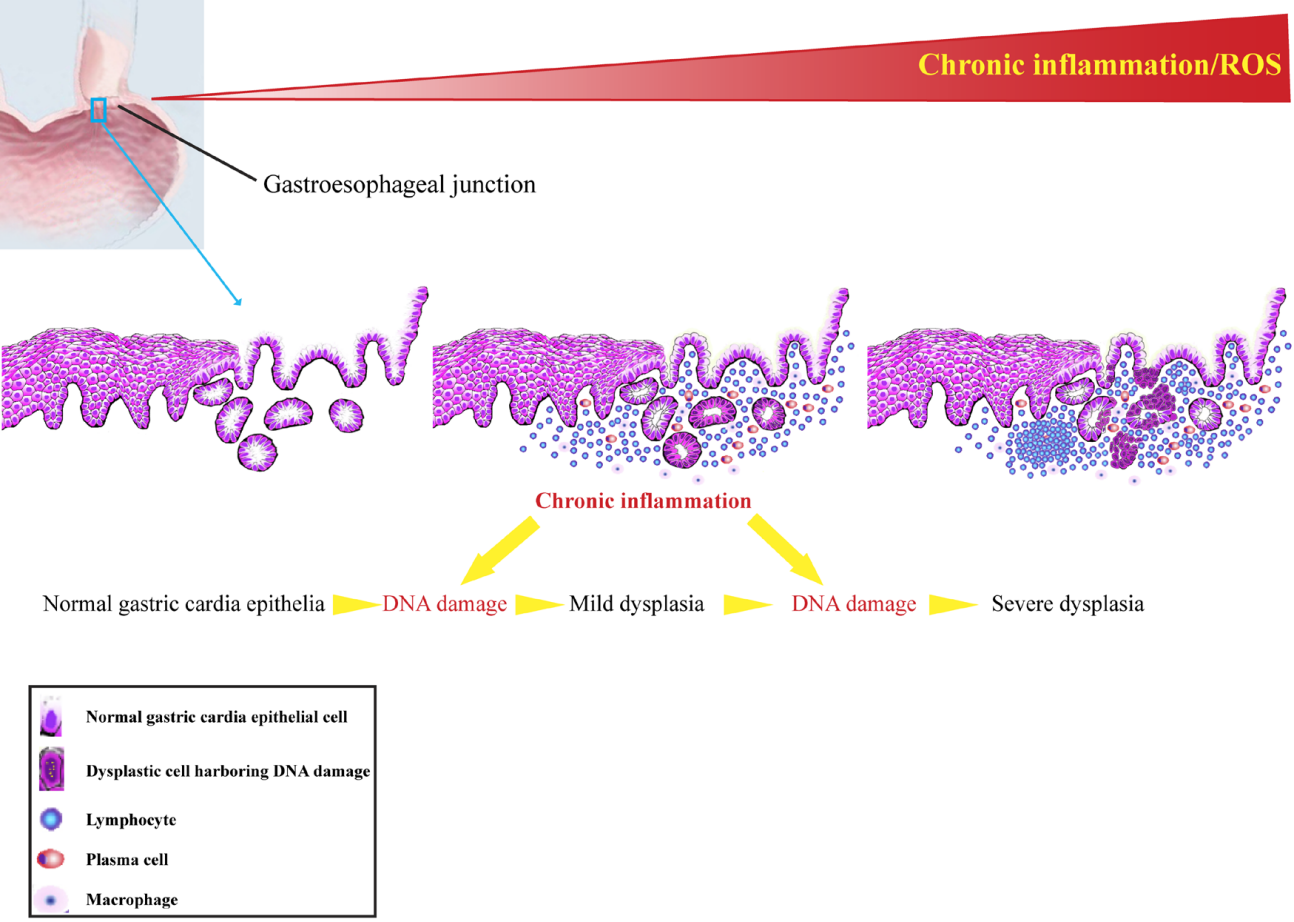
Schematic diagram of persistent chronic inflammation-related DNA damage response contributes to gastric cardia carcinogenesis

Tumor development is a multi-step process in which initially normal cells undergo a succession of intermediate stages in order to reach a fully malignant phenotype, functional changes of series of genes have occurred during this course [31-33]. Chronic inflammation might increase oxidative stress and damage to several biomolecules, including DNA, which may initiate or promote malignant transformation in the affected tissues. During chronic inflammatory process, immune cells, ranging in size, distribution and composition are recruited to the affected areas. These cells produce a variety of cytotoxic mediators, such as ROS and RNS, which were reported to bring about DNA damage [34]. DNA double-strand breaks (DSBs) are the most serious form of DNA damage and are difficult to repair accurately [35]. Accumulated DNA damage gives rise to mutagenesis and chromosomal rearrangements, contributing to carcinogenesis via genomic instability [36]. Phosphorylated H2AX is considered to be a reliable biomarker of DSBs [37]. Our previous study showed that CagA+ *H. pylori* could induce strong DNA damage in human immortalized esophageal epithelial cells [38]. Moreover, increased levels of γH2AX were found in colonic mucosal specimens from patients with ulcerative colitis compared to control samples [39]. In this present study, our results demonstrated that a higher level of DNA damage response was detected in epithelia with severer inflammation, suggesting of a close association between chronic inflammation and DNA damage response. The elevated DNA damage response levels could be a result of increased production of RONS from infiltrating inflammatory cells. Additionally, we also detected higher level of DDR in tissues with different degrees of intraepithelial neoplasia compared to normal epithelia. Besides, several DNA damage response markers have been reported to be activated by chronic inflammatory stress [40]. The remarkable accumulation of DNA damage response in samples with intraepithelial neoplasia strongly indicates that unrepaired DNA damage in these precancerous tissues may be a main driving force of gastric cardia tumorigenesis. Hence, DNA damage response may be selected as a promising biomedical marker for indentifying potential carcinogenesis in individuals with chronic inflammation in the gastric cardia. Our results suggest a hypothesis (Figure 4) that increased gastric cardia epithelial cells harboring DDR in the milieu of chronic inflammation may precipitate carcinogenesis. As such, critical genes involving in cell proliferation and differentiation are probably affected due to the accumulated DNA damage, and prompt neoplastic transformation.

It has been reported that inflammation-related NF-κB pathway can activate mTOR pathway and thus regulate DDR [41], supporting our findings that higher level of DDR was frequently observed in inflamed tissues. Rapamycin, a clinically approved drug, was suggested to be potentially used to decrease DDR [42], this is also a promising therapeutic target for early intervention, for our data demonstrated that higher level of DDR presented in dysplastic tissues compared to the normal tissues.

In conclusion, this study showed that DNA damage response was detectable in gastric cardia mucosa with chronic inflammation. This DNA damage response correlated with the severity of chronic inflammation as well as the development of precancerous mucosal changes. Thus, we speculate that DNA damage response might serve as a critical link between chronic inflammation and gastric cardia malignancy through its high potential for causing DNA mutations, which is a main driving force of gastric cardia carcinogenesis. DNA damage response, therefore, might be considered as a promising diagnostic marker for identification of potential malignant transformation in gastric cardia biopsies at early stage, particularly in inflamed tissues. Given the relatively small sample size in our current study, future large-sample investigations should be performed to further evaluate the application value of DNA damage response in clinical setting.

## MATERIALS AND METHODS

### Patients and sample collection

A total of 50 paired tissue samples from GCC patients with no prior radio- or chemotherapy were collected from Cancer Hospital of Shantou University Medical College from January 2010 to December 2012. For each patient, tumor-surrounding non-malignant tissues (within 2 cm from tumor) and distant non-malignant tissues (more than 5 cm away from tumor) were analyzed. The total 100 samples were categorized as normal, low-grade intraepithelial neoplasia (LGIN) and high-grade intraepithelial neoplasia (HGIN). All samples were fixed, dehydrated and paraffin embedded, and their pathological features and diagnoses were verified by two pathologists with hematoxylin and eosin staining. Baseline data of the 50 GCC patients and sample profile were shown in Table 1. This study was approved by Ethics Committee of Shantou University Medical College, and informed consents were obtained from patients or their families.

**Table 1:** Summary of the baseline data of GCC patients and the examined samples

Variable	No.
Age, yrs (range), n=50	62.3±8.1 (46-76)
Sex, n=50	
Male (%)	38 (76)
Female (%)	12 (24)
Histological condition, n=100	
Normal/hyperplasia (%)	47 (47)
LGIN (%)	48 (48)
HGIN (%)	5 (5)
Chronic inflammation, n=100	
Normal (%)	23 (23)
Mild (%)	30 (30)
Moderate (%)	25 (25)
Severe (%)	22 (22)

LGIN: low-grade intraepithelial neoplasia; HGIN: high-grade intraepithelial neoplasia.

### Immunohistochemistry

Paraffin-embedded samples were serially sectioned at 4 μm, mounted on gelatin-coated slides, dried at 60°C for 3 h. The sections were deparaffinized in xylene, rehydrated in a descending series of ethanol solutions, and then treated with 3% hydrogen peroxide for 15 min to quench endogenous peroxidase activity. Antigen retrieval was performed by microwaving the sections immersed in citric acid buffer (10 mM, pH 6.0) for 20 min, followed by 1 h incubation at room temperature. To block non-specific staining, we incubated sections with 10% normal goat serum. Next, primary antibodies for γH2AX and phospho-ATM Ser1981 were added, and the slides were incubated at 4°C overnight. After washing with PBS, corresponding secondary antibodies were added for incubation at 37°C for 30 min before reaction with diaminobenzidine and counterstaining with hematoxylin. Slides were then dehydrated and mounted. Positive controls were used for each of the antibodies (breast cancer tissues for γH2AX and phospho-ATM Ser1981), while in the negative controls the PBS was substituted for the primary antibody.

Images were captured using a Leica IM50 microscope at ×400, and five different fields for each index were selected in each sample. The number of positive nuclei and total nuclei in images was then counted with the application of Image Pro Plus (IPP) 6.0 software, and the corresponding labeling index (percentage) was computed as positive nuclei/total nuclei × 100%.

### Western blot

Tissues were lysated in RIPA lysis buffer supplemented with protease inhibitor (Beyotime, China). Protein samples (50 μg) were denatured, separated by SDS-polyacrylamide gel electrophoresis and transferred to PVDF membranes. After blocking in TBS with 5% BSA, the membranes were incubated with primary antibodies against γH2AX (1:1000, Cell Signalling) and ACTIN (1:1000, Sigma Aldrich) overnight at 4°C. After washing, the membranes were incubated with corresponding secondary antibodies at room temperature for 1 h. The blotted membranes were washed and visualized using ECL reagents following the manufacturer's instructions.

### Statistical analysis

All statistical analyses were performed using Statistical Package for Social Sciences (SPSS) 13.0 (SPSS Inc., Chicago, IL USA). One-way analysis of variance was used for labeling index analysis in multiple groups. The difference of labeling index between two groups was evaluated with *t*-test. A two-tailed *P*≤0.05 was considered statistically significant. All reported *P* values were two-sided.
